# The Foreign Journals: Animal Chemistry and Pharmacy

**Published:** 1841-10

**Authors:** 


### ANIMAL CHEMISTRY AND PHARMACY.

### *On the Chemical Composition of the Brain.* By M. Fremy.

According to the analyses of M. Fremy the human brain is formed of a considerable quantity of water, and of matter insoluble in æther, which he calls albuminous matter. It contains also a part soluble in æther which is formed by three matters: 1, white matter, which he calls cerebric acid, he having discovered it to possess well-marked acid properties; 2, a liquid fatty matter, which has the composition and all the properties of the oleine of human fat; 3, cholesterine; also very slight and variable traces of oleaic and margaric acids, and cerebrate of soda. The albuminous matter of the brain has the property of transforming oleine into oleaic acid.

*Journal des Connaissances Médico-Chirurgicales. Janvier*, 1841.

---

### *On the identical Composition of Fibrin and Albumen.* By M. Liebeg, of Giessen.

M. Liebeg states that he has been able to dissolve pure fibrin in a saturated solution of nitre, at a temperature from 50 to 56 degrees. The fibrin becomes gelatiniform, merely leaving a few flocculi, which are insoluble. The filtered fluid possesses all the properties of albumen, and the composition of both is exactly the same. It is as follows:

| | |
|---|---|
| Carbon | 48 |
| Hydrogen | 74 |
| Nitrogen | 14 |
| Oxygen | 11 |

M. Liebeg has also precipitated albumen under the form of globules, by adding a sufficient quantity of water to serum which had been neutralized by an acid, and he has likewise obtained fibrin from the blood-globules, by the proceeding described by M. Denis. Lastly, by adding a little caustic potash to albumen, M. Liebeg has precipitated it under the form and with the properties of casein, by means of alcohol.

*Gazette Médicale de Paris.* 3 *Avril*, 1841.

